# PreserFlo MicroShunt Tube Embedded in the Iris: Successful Treatment Using Argon and Neodymium-Doped Yttrium Aluminum Garnet (Nd:YAG) Lasers and Laser Iridotomy for Recurrence Prevention

**DOI:** 10.7759/cureus.79850

**Published:** 2025-02-28

**Authors:** Azusa Yamagishi, Takayuki Baba

**Affiliations:** 1 Ophthalmology, Chiba University, Chiba, JPN

**Keywords:** argon laser, intraocular pressure (iop), iris embedding, laser iridotomy, minimally invasive glaucoma surgery, nd:yag laser, pigmentary glaucoma, poly(styrene-b-isobutylene-b-styrene) (sibs), preserflo microshunt, tube occlusion

## Abstract

The PreserFlo MicroShunt (PMS) (Santen Pharmaceutical, Co., Ltd., Osaka, Japan) procedure is classified as minimally invasive glaucoma surgery (MIGS) and is a type of tube shunt surgery. Tube occlusion in PMS is a rare but significant complication that requires intervention unless caused by temporary thrombi. This report describes a rare case where a PMS tube became completely embedded in the iris, and the condition was successfully managed with the combined use of argon and neodymium-doped yttrium aluminum garnet (Nd:YAG) lasers. A 54-year-old woman with pigmentary glaucoma in her right eye underwent trabeculectomy but failed to achieve adequate intraocular pressure (IOP) control. PMS implantation was performed approximately five months after the initial surgery. Seven months post-implantation, the entire PMS tube became embedded in the iris, leading to IOP elevation. Argon and Nd:YAG lasers were used to relieve the occlusion, followed by laser iridotomy around the tube tip to prevent re-occlusion. The IOP was well-controlled immediately after the laser procedure and remained stable for three months postoperatively, with no recurrence of occlusion or corneal endothelial damage. The combined use of argon and Nd:YAG lasers appears to be a promising and safe treatment option for managing iris-related PMS occlusion and preventing recurrence.

## Introduction

The PreserFlo MicroShunt (PMS) (Santen Pharmaceutical Co., Ltd., Osaka, Japan) is a tube device made of poly(styrene-b-isobutylene-b-styrene) (SIBS) with an internal diameter of 70 μm and a total length of 8.5 mm. PMS creates an aqueous humor outflow pathway from the anterior chamber to the subconjunctival space, which forms a filtration bleb similar to that in trabeculectomy (TLE). The Food and Drug Administration and the American Glaucoma Society classify PMS as a minimally invasive glaucoma surgery (MIGS) device, specifically a subconjunctival ab externo transscleral filtration device [1]. Unlike TLE, PMS does not require postoperative interventions, such as laser suture lysis, and has a lower risk of hypotony-related complications [2,3]. However, its long-term intraocular pressure (IOP)-lowering efficacy is slightly lower than that of TLE [3].

Tube occlusion following tube shunt surgery is a rare but critical complication that can lead to sudden elevation of IOP and affect long-term IOP control. Reported treatments for tube tip occlusion by the iris include argon laser-induced iris contraction and surgical repositioning. Here, we report a case in which the PMS tube was completely embedded in the iris approximately seven months after implantation, which led to IOP elevation. The patient was successfully treated with an argon laser and a neodymium-doped yttrium aluminum garnet (Nd:YAG) laser, thus achieving a significant reduction in IOP.

## Case presentation

A 54-year-old woman was referred to our hospital with poor IOP control in her right eye. At her initial visit, the corrected visual acuity was 0.15 in the right eye and 1.2 in the left eye. Despite intensive treatment with latanoprost, timolol and brinzolamide, brimonidine, and ripasudil, the IOP was 48 mmHg in the right eye and 14 mmHg in the left eye. Corneal endothelial cell density could not be measured because of corneal edema. Gonioscopy revealed dense pigment deposition throughout the trabecular meshwork in the right eye, leading to a diagnosis of pigmentary glaucoma. The patient underwent selective laser trabeculoplasty (SLT) (107 shots, 0.9 mJ; Selecta Duet, Lumenis, Tokyo, Japan), which lowered her IOP to 14 mmHg the following day. However, her IOP rose to 56 mmHg four days later, prompting TLE with 0.04% mitomycin-C (MMC) (Mitomycin-C "Kyowa," Kyowa Kirin Co., Ltd., Tokyo, Japan) performed in the superior temporal quadrant. Although her IOP was well-controlled for one month postoperatively, it subsequently increased. Despite undergoing four sessions of needle revision without MMC and one procedure with MMC, her IOP remained uncontrolled, leading to the decision to perform PMS implantation. Corneal endothelial cell density was 2285 cells/mm² immediately after TLE, but it was 1304 cells/mm² before PMS insertion due to repeated needling.

Approximately five months after the patient's initial visit, PMS implantation was performed in the superior nasal quadrant of her right eye. The conjunctiva was incised, and 0.04% MMC was applied to Tenon’s capsule and sclera for three minutes, followed by thorough irrigation with a balanced saline solution. An entry incision was made 3 mm posterior to the limbus using a double-step knife to create a scleral tunnel into the anterior chamber, through which the PMS was inserted. The conjunctiva and Tenon’s capsule were sutured with 10-0 nylon. Postoperatively, the PMS was correctly positioned anterior to the iris.

**Figure 1:**
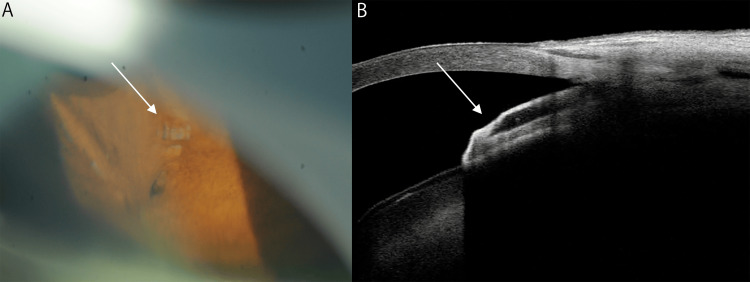
Gonioscopy and anterior segment optical coherence tomography (AS-OCT) findings seven months post-implantation of the PreserFlo MicroShunt (PMS) (A) Gonioscopy revealed that the PMS tube was partially embedded in the iris, except for the tip of the tube. The white arrow indicates the tube tip. (B) AS-OCT demonstrates the PMS tube embedded in the iris. The white arrow indicates the tube tip, whose opening was not observable on AS-OCT.

The patient’s postoperative course was initially favorable, with no complications observed during the early recovery period. However, seven months after implantation, most of the PMS tubes (excluding the tip) became embedded in the iris (Figure 1). As the tube opening remained patent and her IOP was stable at 8 mmHg, the patient was monitored without further intervention.

Five days later, she presented with blurred vision in her right eye. Her IOP had increased to 61 mmHg, while the PMS opening was completely occluded by the surrounding iris tissue.

**Figure 2:**
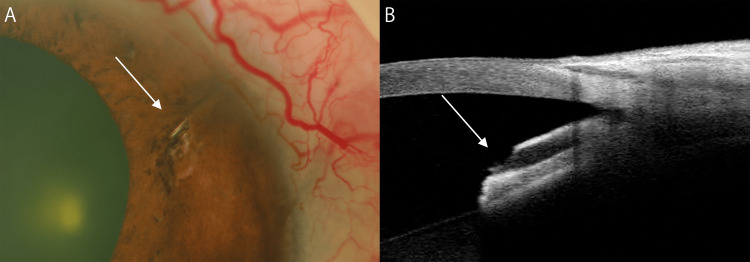
Slit lamp and anterior segment optical coherence tomography findings five days after the observations shown in Figure 1 and following the first laser treatment for PreserFlo MicroShunt embedding (A, B) The white arrow indicates the tip of the tube, which was reopened by argon and Nd:YAG lasers.

We first applied an argon laser (Integre Pro Scan, Ellex, Inc., Tokyo, Japan; 561 nm, spot size 200 μm, power 200 mW, duration 200 ms) six times at the presumed location of the PMS tip to stretch the iris tissue. Next, an Nd:YAG laser (Selecta Duet; 1 mJ) was applied six times to remove the iris tissue overlying the tube opening (Figure 2). We did not use any specific technique for pupil management during the laser treatment. No damage to the PMS was observed intraoperatively. Following the procedure, the tube opening was restored, and the IOP decreased to 10 mmHg. Betamethasone sodium phosphate eye drops were prescribed hourly on the day of the procedure and four times daily thereafter.

**Figure 3:**
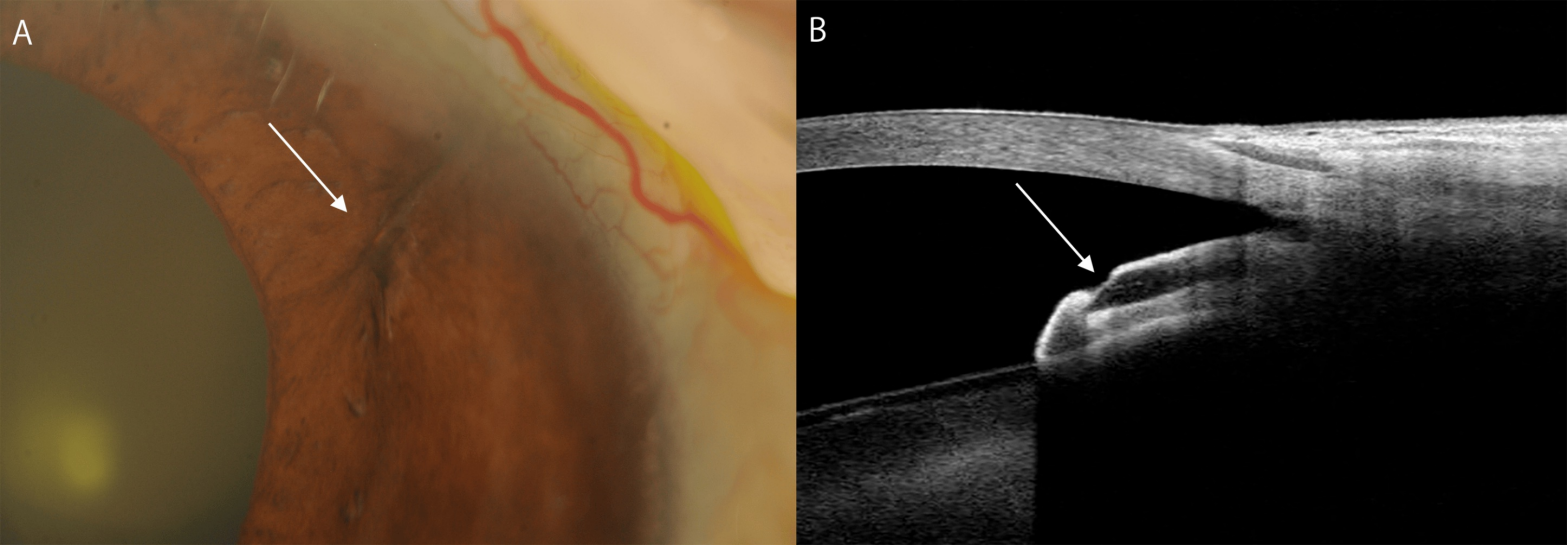
Slit lamp and anterior segment optical coherence tomography findings 14 days after the first laser treatment (A, B) The white arrow indicates the PreserFlo MicroShunt tip, which was partially covered by the iris again before the second laser treatment.

**Figure 4:**
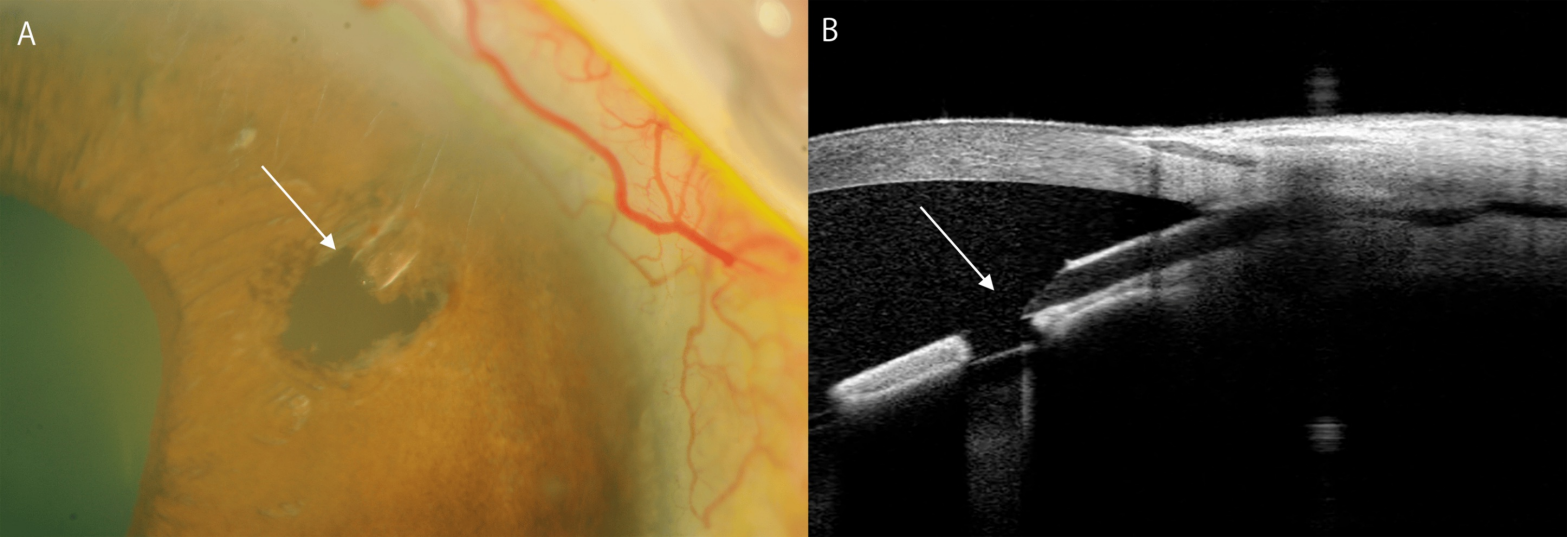
Slit lamp and anterior segment optical coherence tomography findings after the second laser treatment (A, B) The white arrow indicates the fully exposed PreserFlo MicroShunt tip, and the surrounding iris tissue was penetrated by argon and Nd:YAG lasers.

Fourteen days later, the patient's IOP remained stable at 8 mmHg. However, the iris began to cover the PMS tip partially, necessitating another laser procedure(Figure 3). This time, argon laser treatment was applied to the surrounding iris tissue: 30 shots (561 nm, spot size 200 μm, power 200 mW, duration 200 ms) to stretch the iris tissue and 30 shots (561 nm, spot size 50 μm, power 1000 mW, duration 20 ms) to partially ablate the iris tissue, along with 40 Nd:YAG laser shots (1.0 mJ) to penetrate the iris tissue. These lasers were applied in combination until the PMS tip was fully exposed and the surrounding iris tissue was penetrated (Figure 4). Moreover, no melting or damage of the PMS tube was observed. We did not use any specific pupil management technique even when the laser treatment was repeated. Betamethasone sodium phosphate was prescribed, following the same protocol as that used after the first laser procedure.

**Figure 5:**
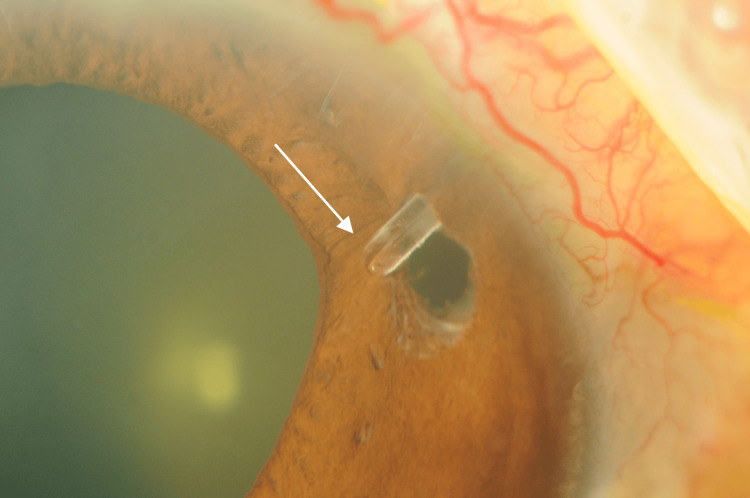
Slit lamp findings three months after the second laser treatment (A, B) The white arrow indicates the patent PreserFlo MicroShunt tip.

Three months after the second laser treatment, the tube remained patent, and the IOP remained stable without recurrence (Figure 5). Corneal endothelial cell density remained unchanged at 1358 cells/mm² before the procedures and 1379 cells/mm² afterward.

## Discussion

Tube occlusion during PMS implantation has been reported in 0%-3% of cases, making it a rare complication [2,4,5]. Furthermore, previously reported cases of iris-related occlusion involved blockage confined to the tube tip [6], whereas cases such as the present case in which the entire tube inserted into the anterior chamber becomes embedded in the iris are exceedingly rare. Iris occlusion after tube shunt surgery can occur when the tube comes into contact with the iris [6,7]. In the present case, the PMS tube may have been in contact with the iris. However, iris contact with the PMS itself is not particularly rare, and its likelihood of leading to occlusion is low. A study observed long-term contact of PMS with the iris in 13% of cases. Tube reinsertion was unnecessary in these cases, and there was no significant impact on long-term outcomes [8]. While avoiding excessive proximity to the iris during PMS implantation may help prevent occlusion, placing the PMS excessively close to the corneal endothelium can lead to endothelial cell loss [9], and direct contact with the corneal endothelium can cause endothelial damage [10]. Therefore, careful positioning of the PMS to avoid both the iris and the corneal endothelium is essential.

In this case, we applied argon and Nd:YAG lasers to the occluded area to reopen the tube and subsequently performed laser iridotomy (LI) around the tube tip to prevent recurrence. Similar techniques for tube shunt surgeries other than those involving PMS have not been reported. Regarding the treatment of PMS tube occlusion, three cases have been reported: one involving Nd:YAG laser treatment for iris occlusion, another where surgical removal of iris fibers was performed for occlusion caused by the iris tissue, and a third where Nd:YAG laser was applied inside the tube to treat fibrin occlusion [6,7,11]. In other types of tube shunt surgery, occlusions caused by the iris, thrombi, vitreous, or lens capsule have been treated with Nd:YAG lasers, argon lasers, or surgery [5,12-14]. Attempts to resolve iris-induced occlusion have often been unsuccessful [6,14]. For iris occlusion involving the XEN45 Gel Stent (AbbVie Inc., North Chicago, IL, USA ), an argon laser was used to contract the surrounding iris tissue in a C-shaped pattern, which successfully reopened the occluded tube [13,14]. In this case, the entire tube was embedded in the iris, thus requiring a treatment approach different from previously reported methods. A combination of argon and Nd:YAG lasers was used to achieve IOP reduction without tube reinsertion. Additionally, LI was performed to prevent recurrence, which resulted in favorable outcomes.

The risks of using argon and Nd:YAG lasers for PMS treatment remain unclear. Nd:YAG lasers have been used in previous cases of PMS occlusion [7]; however, no reports have documented argon laser use for PMS occlusion. For XEN occlusion, the argon laser targets the surrounding iris tissue, not the tube [13,14]. During argon laser treatment, we carefully avoided the PMS tube as a direct target. Instead, the laser was applied to the iris tissue either covering or adjacent to the tube.

Argon laser energy is strongly absorbed by pigmented tissues such as the iris, whereas its absorption by transparent tissues including the cornea or PMS is minimal [15]. Consequently, because of its material properties, the PMS absorbed minimal laser energy. However, the heat generated in the adjacent iris tissue was likely transferred to the tube. Biological tissues are known to coagulate at 60-80 °C and vaporize above 100 °C [16].

The SIBS material of the PMS is a thermoplastic elastomer that lacks a defined melting point. Its physical properties are determined by its glass transition temperature, weight loss, and thermal decomposition temperature [17]. Although detailed studies on the thermal resistance of SIBS are limited, its thermal decomposition temperature is approximately 386 °C [18]. Brief bubble formation was observed during argon laser treatment. Given that the bubbles were transient, heat transfer to the tube was minimal. No evidence of melting or deformation of the PMS was found. Postoperatively, anterior segment inflammation was mild, and no corneal endothelial cell loss was observed. To date, no severe complications from the laser treatment have been reported.

## Conclusions

Seven months after PMS implantation, we successfully treated a rare case of a tube becoming embedded in the iris using a combination of argon and Nd:YAG lasers. LI was performed around the tube tip to prevent recurrence, and the patient remained stable for three months post-treatment.

The combination of argon and Nd:YAG lasers appears to be a promising treatment option for managing iris-induced PMS occlusion and preventing recurrence. This option should be considered before performing more invasive treatments, such as surgical device replacement. However, careful monitoring is required for potential long-term complications and recurrence.
